# A Review on the Effects of Soccer Small-Sided Games

**DOI:** 10.2478/v10078-012-0049-x

**Published:** 2012-07-04

**Authors:** Marco Aguiar, Goreti Botelho, Carlos Lago, Victor Maças, Jaime Sampaio

**Affiliations:** 1 CIDESD Research Unit, Sport Sciences Department, University of Trás-os-Montes e Alto Douro, Portugal.; 2 CERNAS Research Unit, Coimbra College of Agriculture, Polytechnic Institute of Coimbra, Portugal.; 3 Faculty of Education and Sports Sciences, University of Vigo, Campus Pontevedra, Spain.

**Keywords:** soccer, training, conditioning, heart rate, players, technique, perceived exertion

## Abstract

Over the last years there has been a substantial growth in research related to specific training methods in soccer with a strong emphasis on the effects of small-sided games. The increase of research in this topic is coincident with the increase of popularity obtained by specific soccer conditioning, which involves training players to deal with soccer match situations. Given the limited time available for fitness training in soccer, the effectiveness of small-sided games as a conditioning stimulus needs to be optimized to allow players to compete at the highest level. Available studies indicate that physiological responses (e.g. heart rate, blood lactate concentration and rating of perceived exertion), tactical and technical skill requirements can be modified during small-sided games by altering factors such as the number of players, the size of the pitch, the rules of the game, and coach encouragement. However, because of the lack of consistency in small-sided games design, player fitness, age, ability, level of coach encouragement, and playing rules in each of these studies, it is difficult to make accurate conclusions on the influence of each of these factors separately.

## Introduction

Soccer is probably the most popular sport in the world. Despite its universal nature and its formal history extended back over a hundred years, there are still many uncertainties concerning its multidimensional requirements (physiological, psychological, biomechanical) and therefore uncertainties when planning for optimal training and conditioning. In fact, this game is very complex because the pitch is substantially large (approximately 100 × 60 m), the ball is controlled with the feet and head and there may be interactions within eleven teammates and between eleven opponents, almost all with different roles in the game.

Such complexity is currently addressed in training sessions by using specific tasks with the goal of reducing interactions and increasing the ratio of players’ participation in decision making, but preserving basic variability properties from the game (Capranica et al., 2001; Gabbett, 2002; 2006; Jones and Drust, 2007; Rampinini et al., 2007; Frencken and Lemmink, 2008; Hill-Haas et al., 2009c; 2010; Katis and Kellis, 2009). These tasks are known as small-sided games (SSG) and its study is currently one of the most addressed topics in soccer contemporary research (Hill-Haas et al., 2009c, 2010).

In high performance sports it has been well documented that the maximum benefits are achieved when the training stimuli are similar to competitive demands (Bompa, 1983). In order to reproduce the physical, technical and tactical requirements of real match play (MacLaren et al., 1988; Miles et al., 1993; Hoff et al., 2002; Reilly and White, 2004; Sassi et al., 2004), coaches often use SSG in their training programs.

SSG started as an optimal task to optimize training time by fulfilling the broad range of fitness requirements without compromising skill performance and decision-making. Therefore, they are used extensively to improve physical fitness levels and also technical and tactical performance in a wide variety of soccer codes (Balsom, 1999; Drust et al., 2000; Gabbett, 2002; Nurmekivi et al., 2002; Bangsbo, 2003; Reilly and Gilbourne, 2003; Gamble, 2004; Eniseler, 2005; Gabbett, 2005; Reilly and White, 2005; Sainz and Cabello, 2005; Sassi et al., 2005; Rampinini et al., 2007; Aguiar et al., 2008; Duarte et al., 2009, Hill-Haas et al., 2008, 2009a,b,c, 2010).

The intensity of these soccer-specific training drills with the ball can be affected or manipulated to provide different physical, technical and tactical responses by several factors, such as, the number of players involved, the size and the shape of the pitch, the duration of exercise and rest periods, the rules of the game, coach encouragement, the availability of balls or by the way of scoring points (Bangsbo, 1994; Balsom, 2000; Hill-Haas et al., 2009a). A better understanding of the influence of modifying those variables on SSG will assist coaches in controlling the training process with players.

Recent literature has been focused on the physiological and technical aspects of SSG (Impellizzeri et al., 2006; Tessitore et al., 2006; Jones and Drust, 2007; Dellal et al., 2008; Frencken and Lemmink, 2008; Castagna et al., 2009; Couts et al., 2009; Hill-Haas et al., 2009b; Katis and Kellis, 2009). Secondly, the emphasis has been placed on the restrictions of the task that may have effects on the physiological responses to SSG (Rampinini et al., 2007; Kelly and Drust, 2008; Hill-Haas et al., 2008; 2009a, c; 2010).

## Research

Soccer constitutes an open skill team sport characterized by performance under variable conditions and it can be expected that different aspects of performance might vary according to situational circumstances (Tessitore et al., 2006). Coaches can administer a large variety of drills by changing the size and the shape of the playing area, the number of players, the rules and drill duration. At present, training sessions are focused on enhancing both physiological and technical-tactical aspects of play through general conditioning bouts and training drills.

In the last years, with the increased use of SSG as a training method, the scientific community devoted greater attention to them. In fact, we found recent studies with conclusions which suggest that physiological responses (e.g. heart rate, blood lactate concentration and rating of perceived exertion) and technical/skill requirements can be modified during SSG in soccer by altering factors such as the number of players, the size of the pitch, the rules of the game, and coach encouragement (Casamichana and Castellano, 2010; Grant et al., 1999a; 1999b; Little and Williams, 2006; 2007; Owen et al., 2004; Platt et al., 2001; Impellizzeri et al., 2006; Tessitore et al., 2006; Jones and Drust, 2007; Rampinini et al., 2007; Williams and Owen, 2007; Barbero-Álvarez et al., 2008; Dellal et al., 2008; Gabbett and Mulvey, 2008; Kelly and Drust, 2008; Mallo and Navarro, 2008; Castagna et al., 2009; Coutts et al., 2009; Hill-Haas et al., 2008; 2009a,b,c; 2010; Katis and Kellis, 2009).

This review systematizes the SSG use as a training method and highlights the main restrictions of its application in soccer. All factors affecting the SSG are analyzed separately according to studies conducted in order to understand its importance in the response of athletes to training. Despite this division to facilitate the comprehension of these factors, we must take into account that the interaction may affect the final response of athletes. This review also aims to focus on new features of the SSG, still little studied, and because of its great importance, they deserve the attention of researchers. A deeper understanding about the influence of manipulating those variables on SSG will assist coaches in controlling the training process.

### Number of players

Recent studies have shown that SSG formats with a different number of players elicit different physiological, perceptual, and time-motion characteristics (Aroso et al., 2004; Hill-Haas et al., 2009a; 2010; Katis and Kellis, 2009; Sampaio et al., 2007; Owen et al., 2004; Rampinini et al., 2007). It is also common for coaches to use SSG formats that involve a team playing with a fixed numerical advantage against another team with a fixed numerical disadvantage, as used by Hill-Haas et al. (2010). In general, these studies have shown that SSG formats with fewer players elicit greater heart rate than the larger formats (Hill-Haas et al., 2009a; 2010; Impellizzeri et al., 2006; Katis and Kellis, 2009; Little and Williams, 2006; 2007; Owen et al., 2004; Rampinini et al., 2007). However, some authors reported a different conclusion (Aroso et al., 2004; Dellal et al., 2008; Hill-Haas et al., 2008; Hoff et al., 2002; Jones and Drust, 2007; Sampaio et al., 2007) because no differences were found in heart rate responses between SSG formats. Based on the results presented in the aforementioned studies, different SSG formats elicit different heart rate values (Table 1). The variability between different SSG was confirmed by Hill-Haas et al. (2008) who found a typical error less than 5% across the different SSG results.

Fewer studies have focused on the effect of SSG formats on the lactate threshold. However, these studies are more consensual and showed that SSG formats with fewer players elicit greater lactate thresholds (Hill-Haas et al., 2008; 2009a; Impellizzeri et al., 2006; Rampinini et al., 2007).

The rating of perceived exertion (RPE) to the player number changes is in accordance with those found to heart rate responses. In general, these studies have shown that SSG formats with fewer players elicit greater RPE than the larger formats (Aroso et al., 2004; Hill-Haas et al., 2008; 2009a; 2010; Impellizzeri et al., 2006; Rampinini et al., 2007).

The effect of the SSG formats in the technical requirements was addressed by two studies (Jones and Drust, 2007; Katis and Kellis, 2009), suggesting that the number of players should be carefully considered by coaches in their organization. The authors suggested that SSG with small number of players can deliver a more effective technical training stimulus, since the number of technical actions increases with the decrease of players’ number.

The work-rate profiles of players were also observed taking into account the number of players involved (Jones and Drust, 2007; Hill-Haas et al., 2008; 2009a; 2010). The results found are consensual, with most authors claiming that no significant differences were observed in either total distance covered or the total distance covered by walking or jogging. Nevertheless, there is no consensus at high intensity efforts. Hill-Haas et al. (2010) have not found any differences between the amount of players involved in games. Jones and Drust (2007) suggested that high intensity efforts are increased when the number of players is reduced. This conclusion was firstly supported by Platt et al. (2001). The opposite was suggested by Hill-Haas et al. (2008), when these authors observed in their research that maximal and mean sprint duration and distance were increased with the amount of players involved.

These previous studies have only examined the influence of altering the player numbers on teams maintaining a numerical balance between opposing teams (e.g., 2 vs. 2 players and 4 vs. 4 players). It is usual for coaches to use SSG formats that involve a team playing with a fixed numerical advantage against another team with a fixed numerical disadvantage (e.g., 4 vs. 3 players and 6 vs. 5). It is also common to use SSG formats that involve variable “overload” and “underload” situations, which are achieved using a “floater” player. Hill-Haas et al. (2010) studied the response of athletes to this and they concluded that despite fixed underload teams recording a significantly higher RPE compared with the fixed overload teams, there were no differences in time-motion characteristics and physiological responses. According to these authors, both formats (fixed and variable) may provide a useful variation in SSG training or as a technical-tactical training method for defensive and attacking plays. The possibility of variable formats proving a greater technical load needs to be substantiated by further research. Finally, the use of a floater appears to be more effective in smaller format games and may be appropriate for either maintaining or developing aerobic fitness.

The different conclusions may be due to the fact that the methodology adopted by the authors and the population that served as a sample study were distinct. Another fact that may have led to such different conclusions is the difficulty found to isolate all the factors which can contribute to the physiological and technical response of players. Finally, another possible reason for discrepant results includes the fact that the pitch size masks the effects of variations in the number of players.

### Pitch size

Research has shown that using different pitch dimensions and formats can elicit different physiological and perceptual responses, as well as time-motion activity. However, studies are not consensual on the influence of the pitch size in the physiological response of the players. In the origin of this disagreement is probably the fact that research has been carried using several different pitch sizes (Table 2).

According to Tessitore et al. (2006) coaches can modify training intensity by varying pitch dimension, with smaller individual area having a large impact on metabolic demands of exercise. In this study, the exercise intensity ranged from 61% to 76% of the players maximal oxygen uptake, with lower values for the larger pitch. These results are similar to those obtained by Kelly and Drust (2008), as the authors did not find different heart rate responses between SSG played in three pitch dimensions. On the contrary, Rampinini et al. (2007) and Casamichana and Castellano (2010) found significant differences in heart rate responses between SSG played on pitches with different sizes. Higher HR values during SSG played on a large pitch were registered when compared to medium- and small-sized pitches.

Blood lactate variation due to different pitch sizes suggests that drills played in a bigger pitch resulted in a more aerobic activity with a higher occurrence of intensities up to the lactate threshold (Tessitore et al., 2006; Rampinini et al., 2007). In their study, Tessitore et al. (2006) concluded that 6-a-side drills played on the bigger pitch resulted in a greater aerobic activity with a higher occurrence of intensities up to the lactate threshold (50 × 40 m pitch: 3 min 85%; 8 min: 65%) with respect to the smaller pitch (30 × 40 m pitch: 3 min 50%; 8 min: 39%). Those results were corroborated by Rampinini et al. (2007) who found higher blood lactate values during different small-sided game forms played on a larger pitch than on medium- and small-sized pitches.

RPE have a multifactorial nature, which is mediated not only by physiological but also by psychological factors (Borg et al., 1987; Morgan, 1994). This may cause a large variability among subjects, and is one of the limitations to drawing of conclusions about the effect of the SSG pitch area in the RPE. Only in the studies conducted by Rampinini et al. (2007) and Casamichana and Castellano (2010) addressed the specific effect of pitch dimension on the RPE. The authors found differences between medium and large pitches, both of which resulted in higher RPE ratings relatively to smaller pitches. Analyzing these findings together with those obtained in previous studies not specific about the effect of the play area in the RPE, it seems that increasing the ratio between the area × player reduces player perception of effort in SSG training (Hill-Haas et al., 2009a). Casamichana and Castellano (2010) found that the effective playing time could offer a potential explanation for the differences in the physiological, physical and perceived exertion variables studied in SSG: as the individual playing area was reduced, the frequency of motor behaviors increased, with a concomitant decrease in effective playing time (since a greater number of rule-related interruptions leads to a shorter effective playing time). At the same time, the players cover less ground, spending more time stationary or walking, which leads to a lower physiological workload and lower ratings of perceived exertion.

Some authors also observed the influence of the pitch size in the technical actions and they found no significant differences in the frequency of most actions, such as passing, receiving, dribbling, interceptions or headings (Tessitore et al., 2006; Kelly and Drust, 2008). However, Kelly and Drust (2008) found a high number of shots and tackles in the smaller pitches. This conclusion was supported by the data obtained by Owen et al. (2004). The increase of tackles in smaller SSG pitch sizes may be due to smaller area per player, which causes a greater proximity to the opponents and hence greater physical contact. On the other hand, the increasing number of shots can be justified by the proximity of goals, which can lead soccer players to make more frequent attempts at the goal. According to Kelly and Drust (2008), this would suggest that pitch size should only be carefully considered by coaches in their organization of practice if the drill is required to combine a physical training stimulus with technical work on shooting or if minimal physical contact in training is the objective.

Previous studies allow us to conclude that there is no consensus about the influence of the pitch size on physiological responses of athletes. However, these studies are in agreement stating that this factor has no significant influence on technical demands. The different conclusions reached in the performed studies about physiological demands can be due to different methodologies. For example,

Tessitore et al. (2006) and Rampinini et al. (2007) did not examine the isolated impact of key independent variables, such as exercise type, pitch dimension, coach encouragement. As a consequence, their studies are limited in its ability to clearly differentiate the impact of specific variables on the physiological responses to SSG. Moreover, by altering pitch size we can regulate the athlete’s effort intensity in SSG training. According to reports from players and coaches when the ratio of the playing area and number of players is increased, exercise intensity increases as well (Rampinini et al., 2007). This might be explained by the increased playing area that each player has to cover, which means more displacement and probably movements with higher speed. For example, Balsom (1999) suggested that during four-a-side games, intensity similar to that in three-a-side games could be reached by increasing the playing area.

In general, the variation found in the pitch areas of the same SSG format does not allow us to draw definitive conclusions about the effect of the pitch size on the player’s response. To overcome this problem, sport researchers should define to each SSG form a standard area to perform their studies, and define what should be a small, medium or large area. In our opinion, only with standardized pitch sizes and methodologies we are able to isolate the specific effect of pitch size on athletes from the other factors (e.g. number of players, coach encouragement, etc.).

### Presence of goalkeepers and goals

The presence or absence of a goalkeeper in the SSG has some effect on players’ physiological and technical responses. Mallo and Navarro (2008) suggested that the inclusion of a goalkeeper modified the physiological and tactical behavior of the players. The authors found lower heart rates in the game with goalkeepers than in the two games without goalkeepers. These results were not confirmed by Dellal et al. (2008), who found an increase of 10.7% in residual heart rate in the 8-a-side game with goalkeepers. However, the authors found a lower game intensity when the goalkeepers were present. In accordance with the heart rate results, Mallo and Navarro (2008) found exercise intensities higher in the drill performed without goalkeepers. On the other hand, the same authors found a predominance of medium-intensity activities in the drills with goalkeepers. When the technical parameters were analyzed according to presence of goalkeepers, Mallo and Navarro (2008) found lower frequencies of actions with the ball.

These contradictory results can be explained by the fact that the studies did not specifically address the effect of the presence of goalkeepers in the SSG. The inclusion of a goalkeeper probably changed the physiological and tactical behavior of the players (Mallo and Navarro, 2008) because it is possible that some players are more motivated than others by their presence (Dellal et al., 2008). In fact, the aims of scoring and simultaneously protecting their own goalkeepers may have imposed a greater activity on the soccer players (Allen et al., 1998; Dellal et al., 2008; Spalding et al., 2004; Stolen et al., 2005). When playing with goalkeepers, the players will be probably more organized defensively in order to protect their goal, which had a repercussion in game intensity.

### Absence / presence of goals or mini-goals

The intensity of the soccer SSG can be affected by many factors such as the way of scoring (Bangsbo, 1994; Balsom, 2000; Mallo and Navarro, 2008) and the aim of the game (scoring goals or maintaining ball possession). Despite this fact, we have not found a study about the effect of these variables on the physiological and technical response of players during SSG.

### Presence / absence of coach encouragement

Coach encouragement is referred by many authors as one of the factors that influence the player’s physiological response to SSG (Bangsbo, 1998; Balsom, 1999; Couts et al., 2004; Hoff et al., 2002; Mazzetti et al., 2000; Rampinini et al., 2007). This effect could be very important from a practical point of view because the external motivation provided by coach supervision has been shown to achieve greater gains and training adherence, for example, during resistance training (Couts et al., 2004; Mazzetti et al., 2000; Rampinini et al., 2007).

Despite the recognition of its importance, only Rampinini et al. (2007) addressed these effects and found higher heart rates, blood lactates and RPE when the coaches provided encouragement during the SSG. According to the authors, the mean intensity of all SSG included in their study is within the range classified as “high” by Bangsbo (2003). Based on effect size comparisons, the factor that had the greatest influence on the physiological responses to SSG was encouragement, followed by exercise type and field dimensions.

### Rule changes /task constraints

It is also common for coaches to modify the task constraints of SSG in order to change the physical and technical loads imposed on players. Examples of these SSG modifications include restricting the number of ball touches per player or team, implementing (or not) offside rules, changing players to create superiority or inferiority in confronting teams, or alter goal position in the pitch.

Hill-Haas et al. (2010) studied the influence of rule changes in the time-motion characteristics and physiological responses during SSG played by elite youth players. The author introduced five conditions in the games: 1) offside rule in effect (front one-third zone of the pitch); 2) kick in only (ball cannot be thrown in if it leaves the grid); 3) all attacking team players must be in front 2 zones for a goal to count; 4) alongside, but outside the lengths of each pitch, 2 neutral players can move up and down the pitch but not enter the grid; and, 5) 1 player from each team (“a pair”) completes 4 repetitions of “sprint the widths/jog the lengths” on a 90-second interval. The combination of these conditions constitutes the 4 rules applied to the game (example: rule 1 – respect the condition 1+2).

The results obtained by the authors showed that the artificial rule change which required the players to sprint the widths and jog the lengths of the pitch (condition 5) had a greater effect on the time-motion characteristics (total distance traveled, higher intensity running, and number of sprints) than all other rule modifications. However, this artificial rule change had no influence on blood lactate and RPE. According to the author, one possible reason for this may be a “pacing effect” because the relatively long duration of the game, can reduce the rhythm of play, and thereby the physiological and perceptual load, as a strategy to endure the game (Carling and Bloomfield, 2010).

In contrast, a technical rule change requiring that all players from the attacking team to be in the last two-thirds of the pitch for a score to count (condition 3) increased %HR_max_ and blood lactate in both small and large game formats. Despite the duration of the game, this rule change may not have induced a significant pacing strategy because scoring a goal would not have elicited large increases in total distance covered. These results suggest that technical rules that are related to a team’s chance of scoring a goal may influence the player’s motivation to increase or maintain exercise intensity and therefore enhance the player’s physiological response to SSG. Both the technical and the artificial rule changes used in this study had no effect on RPE. The results obtained by the authors are in contrast to previous studies that reported an increase in blood lactate (player-to-player marking) (Aroso et al., 2004), RPE (maximum of 2 touches on the ball) (Sampaio et al., 2007), and %HR_max_ (pressure half switch) (Little and Williams, 2006) with specific rule changes during SSG.

It seems clear that changes in task constraints SSG playing rules can influence and modify the physiological, perceptual and time-motion responses. However, we suggest that rule changing and variations in technical variables in SSG requires further investigation.

### Continuous vs intermittent training

SSG are both reliable and effective for the technical and tactical development of soccer players. However, few studies have examined how the intensity of SSG can be manipulated to alter training stimulus (Fanchini et al., 2011; Hill-Haas et al., 2009c). In soccer, SSG training is typically completed in the form of “intervals” as opposed to continuous duration play, which is more typical of actual game play. However, we found only two studies that focused on the influence or additional benefits of interval or continuous SSG training. In fact, Hill-Haas et al. (2009c) examined the acute physiological responses and time-motion characteristics associated with continuous and intermittent SSG training regimens. The same conclusion was reached by Fanchini et al. (2011). The authors found a significantly lower heart rate in a bout with 2 minutes duration than in bouts with 4 and 6 minutes. The results suggested that continuous SSG elicited a significantly higher percentage of maximum heart rate response compared with intermittent SSG. The additional rest period between work-bouts during intermittent SSG can be the possible explanation. Other possible reason for these results may be a pacing effect, because the relatively long duration of the game in continuous SSG can lead players to reducing the rhythm of play, and thereby the physiological and perceptual load, in order to endure the game (Carling and Bloomfield, 2010).

The percentage of maximum heart rate response for intermittent SSG is similar to that previously obtained in small-formats (Aroso et al., 2004; Owen et al., 2004; Sampaio et al., 2007). The percentage of maximum heart rate response values found with continuous SSG format are similar to those reported for larger format intermittent games (Little and Williams, 2007; Owen et al., 2004; Rampinini et al., 2007). On the other hand, no significant differences were found in the lactate threshold between intermittent and continuous SSG training. These results are similar to those obtained in other intermittent SSG studies (Aroso et al., 2004; Rampinini et al., 2007).

Fanchini et al. (2011) found no significant differences in RPE responses between the three different bouts (2, 4 and 6 minutes). These results were not confirmed by Hill-Haas et al. (2009c). In their study, the authors concluded that continuous SSG elicited a significantly higher RPE response compared with intermittent SSG. These results suggest that higher RPE values are associated with very short intermittent playing durations, which implies that players are able to perform at very high intensity for only short durations during intermittent SSG training. The RPE values obtained by Hill-Haas et al. (2009c) and Fanchini et al. (2011) during intermittent SSG were lower than those previously reported by Aroso et al. (2004) and Sampaio et al. (2007). A possible explanation is the different intermittent playing durations applied in particular studies.

Hill-Haas et al. (2009c) did not divide the time motion results into intermittent and continuous covered distances. However, players covered greater distance between 13.0 and 17.9 km × h^−1^ and at speeds above 18.0 km × h^−1^ during intermittent SSG when compared with continuous SSG. Additionally, the players completed a higher number of sprints and had a higher sprint ratio in intermittent SSG when compared with continuous SSG. One possible explanation to those results was the additional passive rest period between each interval bouts which may have allowed a greater physiological recovery.

Fanchini et al. (2011) also studied the effect of bout duration on technical performance and significant statistical differences between bouts were not found. However, the authors found a tendency to a decrease in total passes, successful passes and interceptions in the bout with highest duration. This fact may suggest that bouts with largest duration may cause a decrease in technical proficiency.

## Conclusions

In SSG the players experience similar situations that they encounter in competitive matches (Owen et al., 2004). Due to this fact, game-based conditioning using SSG has become a popular method of developing specific aerobic fitness for soccer players (Impellizzeri et al., 2006). Despite the increasing popularity of SSG, not many research projects have examined how the intensity of SSG can be manipulated to alter training stimulus (Hill-Haas et al., 2009c). Research was focused on evaluating physiological, tactical and technical responses of athletes when factors such as a number of players, the size of the pitch, rules of the game, and coach encouragement were modified in SSG. The studies appear to confirm that by altering these factors we can manipulate the overall physiological and perceptual workload.

Across the presented studies, we conclude that by changing factors such as a number of players, pitch size, presence/absence of goalkeeper and goals, coach encouragement and the rules, coaches can manipulate the effect of SSG on players. However, because of the lack of consistency in SSG design, player fitness, age, ability, level of coach encouragement, and playing rules among the studies, it is difficult to make accurate conclusions on the influence of each of these factors separately. Due to this limitation, SSG management requires further investigation. The use of standardized conditions in SSG studies will probably allow a better understanding about the role of individual factors and may help researchers to find more reliable conclusions.

## Figures and Tables

**Table 1:** *Maximal heart rate values according to different* SSG formats

**SSG Format**	**% HR_max_ Range**	**Reference**
1-a-side	75 – 80	Dellal et al., 2008
2-a-side	88 – 91	Hill-Haas et al., 2009a; Little and Williams, 2006
3-a-side	87–90	Katis and Kelis, 2009; Litle and Williams, 2006; Rampinini et al., 2007
4-a-side	85–90	Hill-Haas et al., 2009a; Litle and Drust, 2008; Litle and Williams, 2006; Rampinini et al., 2007
5-a-side	82–87	Hill-Haas et al., 2009c; Litle and Williams, 2006; Rampinini et al., 2007
6-a-side	83–87	Hill-Haas et al., 2009c; Katis and Kelis, 2009; Little and Williams, 2006; Rampinini, 2007

**Table 2:** Ranges of pitch area (m^2^) used in each SSG format.

**SSG format**	**Pitch size used (m^2^)**		**Reference**
	**Min.**	**Max.**	
1×1	100		(Dellal et al., 2008)
2×2	400	800	(Dellal et al., 2008) (Hill-Haas et al., 2009b)
3×3	240	2500	(Rampinini et al., 2007) (Gabbett and Mulvey, 2008)
4×4	240	2208	(Coutts et al., 2009)
5×5	240	2500	(Coutts et al., 2009) (Gabbett and Mulvey, 2008)
6×6	240	2400	(Coutts et al., 2009) (Hill-Haas et al., 2009b)
7×7	875	2200	(Hill-Haas et al., 2009b)
8×8	2400	2700	(Jones and Drust, 2007) (Dellal et al., 2008)
